# Medical students’ self-assessed efficacy and satisfaction with training on endotracheal intubation and central venous catheterization with smart glasses in Taiwan: a non-equivalent control-group pre- and post-test study

**DOI:** 10.3352/jeehp.2022.19.25

**Published:** 2022-09-02

**Authors:** Yu-Fan Lin, Chien-Ying Wang, Yen-Hsun Huang, Sheng-Min Lin, Ying-Ying Yang

**Affiliations:** 1 Department of Medical Education, Clinical Innovation Center, Taipei Veterans General Hospital, Taipei, Taiwan; 2 Department of Critical Care Medicine, Taipei Veterans General Hospital, Taipei, Taiwan; 3 College of Medicine, National Yang Ming Chiao Tung University, Taipei, Taiwan; Hallym University, Korea

**Keywords:** Central venous catheterization, Endotracheal intubation, Medical students, Smart glasses, Simulation training

## Abstract

**Purpose:**

Endotracheal intubation and central venous catheterization are essential procedures in clinical practice. Simulation-based technology such as smart glasses has been used to facilitate medical students’ training on these procedures. We investigated medical students’ self-assessed efficacy and satisfaction regarding the practice and training of these procedures with smart glasses in Taiwan.

**Methods:**

This observational study enrolled 145 medical students in the 5th and 6th years participating in clerkships at Taipei Veterans General Hospital between October 2020 and December 2021. Students were divided into the smart glasses or the control group and received training at a workshop. The primary outcomes included students’ pre- and post-intervention scores for self-assessed efficacy and satisfaction with the training tool, instructor’s teaching, and the workshop.

**Results:**

The pre-intervention scores for self-assessed efficacy of 5th- and 6th-year medical students in endotracheal intubation and central venous catheterization procedures showed no significant difference. The post-intervention score of self-assessed efficacy in the smart glasses group was better than that of the control group. Moreover, 6th-year medical students in the smart glasses group showed higher satisfaction with the training tool, instructor’s teaching, and workshop than those in the control group.

**Conclusion:**

Smart glasses served as a suitable simulation tool for endotracheal intubation and central venous catheterization procedures training in medical students. Medical students practicing with smart glasses showed improved self-assessed efficacy and higher satisfaction with training, especially for procedural steps in a space-limited field. Simulation training on procedural skills with smart glasses in 5th-year medical students may be adjusted to improve their satisfaction.

## Introduction

### Background/rationale

Endotracheal intubation (ETI) and central venous catheterization (CVC) are both frequently used essential procedures during clinical practice. These 2 procedures are indicated in many conditions. Recent studies have revealed that early exposure of medical students to medical procedures can improve their competency, confidence, and even the clinical practice of technical skills [1]. Therefore, it is crucial to provide adequate training for future residents to competently perform essential procedures in their careers.

There are different modalities of simulations, including virtual reality [2], augmented reality [3], or web-based video recording systems, and smart glasses are a simulation modality in medical education that has drawn attention recently [4]. Smart glasses are an emerging technology used in simulation-based medical education, defined as a computerized communicator, usually with a video camera, a voice recorder, a voice input interface, and a display screen [4]. The usage of smart glasses in the training of ETI and CVC procedures for junior trainees was also noted in recent years [5].

### Objectives

The purpose of this study was to examine the self-assessed efficacy of 5th- and 6th-year medical students to perform ETI and CVC procedures with smart glasses, and their satisfaction with the training of ETI and CVC procedures with smart glasses.

## Methods

### Ethics statement

This study received approval from the Ethics Committee (Institutional Review Board) of Taipei Veterans General Hospital (2020-06-004BC). The requirement to obtain informed consent was exempted by the institutional review board.

### Study design

This was a non-equivalent control-group pre-and post-test study. This article was described according to the Transparent Reporting of Evaluations with Nonrandomized Designs statement, available at https://www.cdc.gov/trendstatement/index.html.

### Participants

Over 15 months, 145 medical students in their 5th and 6th years participating in clerkships at Taipei Veterans General Hospital were enrolled in this study between October 2020 and December 2021. A workshop for ETI and CVC was held for 6th-year medical students who underwent rotations in critical care units, and 5th-year medical students were also invited to attend the workshop voluntarily. The inclusion criterion was students who voluntarily attended the workshop, and their attendance or performance in the workshop would not influence their clinical score on the rotation. There were no exclusion criteria.

### Intervention

This study was conducted at a medical center and teaching hospital in Northern Taiwan. Hundreds of medical students receive clerkship, internship, and clinical skills training at this hospital every year. In Taiwan, the 6-year medical education program was implemented in 2013, and medical students take part in clerkships during their 5th and 6th years [6]. In the 5th year of the program at this institution, medical students took a preclinical training course first and then underwent 3-month rotations in internal medicine, pediatrics and gynecology, and surgery, respectively. In the 6th year of the program, they participated in rotations in critical care units and the emergency department. They could have had clinical exposure to ETI and CVC procedures during their rotations. Therefore, a standardized simulation-based curriculum of these procedures must provide deliberate practice and assessment for medical students. After a field study to investigate 5th- and 6th-year medical students’ requirements for improving clinical skills, we found that they were interested in essential procedures such as ETI and CVC, which were less available for them to practice in clinical rotations. Therefore, the workshop was opened to better enhance their understanding of lifesaving but risky skills in their future.

**Fig. 1.**
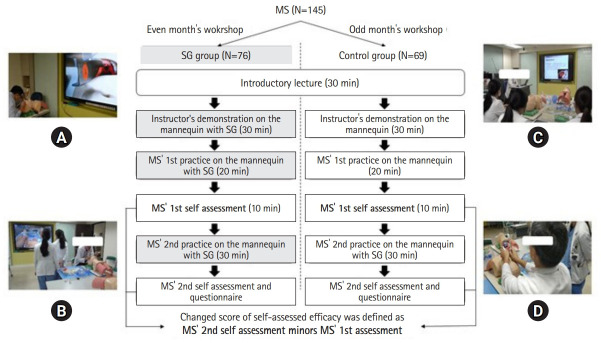
Workshop protocol for smart glasses and control groups. Images of the practice in the workshop included the instructor’s demonstration in the smart glasses (SG) group (A), medical students (MS)’ practice in the SG group (B), introductory lecture by the instructor (C), and the instructor’s demonstration in the control group (D). Source: Professor Ying-Ying Yang. The corresponding author, Dr Ying-Ying Yang, is the owner of the photos in Fig. 1.

During the workshop, the students received an introductory lecture, practice, and assessment for 120 minutes in total (Fig. 1). Firstly, they received an introductory lecture for 30 minutes, and then the instructor would demonstrate with either smart glasses on the mannequin or only the mannequin for 30 minutes, depending on the month in which the workshop was held. In the smart glasses group, to evaluate their understanding of the essential skills of ETI and CVC, students practiced for 20 minutes after the instructor’s demonstration and completed the first self-assessment by wearing smart glasses. After viewing the smart glasses-displayed and gross-detail procedural skills of students, the instructor then gave feedback to each student for 10 minutes. The students then practiced again for 30 minutes and completed a second self-assessment checklist and a questionnaire regarding their satisfaction with the learning experience.

### Assignment methods of the experimental and control group

In odd-numbered months (e.g., 1, 3, 5), medical students attending rotations in intensive care units were assigned to the control group and those attending rotations in intensive care units during even-numbered months (e.g., 2, 4, 6) were assigned to the smart glasses group. Medical students in the control group received demonstrations and practiced on the mannequin only, while those in the smart glasses group received demonstrations and practiced on the mannequin with smart glasses (Supplement 1).

As shown in Fig. 1, there were 8-9 students in the monthly ETT and CVC workshops. In each of 2 parallel skill rooms, 4–5 students were led by 2 similarly experienced instructors with consensus for training and assessment. Each student had 20–30 minutes to practice ETT and CVC on the different mannequins (with and without smart glasses) one by one.

### Blinding (masking)

There was no blinding of the intervention to participants.

### Outcome variables

The outcomes included students’ pre-intervention scores for self-assessment, the change in the score of self-assessed efficacy (the post-intervention score of self-assessment minus the pre-intervention score (Fig. 1), and students’ satisfaction with the training tool, instructor’s teaching, and workshop.

### Data sources/measurement

The examiners collected the students’ self-assessed efficacy and satisfaction program through online google survey. Participants’ response is available at Dataset 1. All variables were recorded in an Excel spreadsheet (Microsoft Corp., Redmond, WA, USA). We used self-assessment checklists (Supplements 2, 3) and satisfaction questionnaires (Supplement 4) to examine medical students’ learning efficacy and their perception of the training. Four statements of the procedural steps in ETI and CVC were included in each checklist (Supplements 2, 3) to evaluate the students’ self-assessed efficacy.

### Validity and reliability of self-assessment checklists and satisfaction questionnaire

For the content validity of each statements in checklists of ETI and CVC and satisfaction questionnaire, an examination by 4 experts revealed content validity index values ranging from 0.78 to 0.83 as shown in Supplements 5 and 6. To assess the reliability of each statement in checklists of the ETI and CVC and the satisfaction questionnaire, the Cronbach α coefficient ranged from 0.70 to 0.89. Additionally, the intraclass correlation coefficients, which were used to assess the agreement among experts on the checklists and satisfaction questionnaire, showed values of 0.78 and 0.75, respectively, indicating good reliability.

### Bias

No bias was found in the study scheme.

### Study size

Sample size calculation was conducted with G*Power ver. 3.1.9.4 (Heinrich-Heine-Universität Düsseldorf, Düsseldorf, Germany; http://www.gpower.hhu.de/), based on the independent sample Student t-test, a 2-tailed alpha of 0.05, power (1-β) of 0.80, and medium effect size (Cohen d) of 0.5 [7]. The result showed that a sample size of around 64 per group was required. Our study included 69 students in the control group, and 76 students in the smart glasses group.

### Unit of analysis

The unit of analysis was the same as the unit of assignment.

### Statistical methods

For students’ pre-intervention scores and changes in scores for self-assessment and satisfaction, a normality test was done, and all the data were normally distributed. We used the Pearson chi-square test to compare categorical variables of the baseline characteristics, and the independent t-test to compare continuous variables. Statistical significance was considered for P-values <0.05. Data were analyzed using SAS software version 9.4 (SAS Institute Inc., Cary, NC, USA). Data were expressed as mean±standard deviation (95% confidence interval).

## Results

### Participants’ baseline data and baseline equivalence

A total of 145 medical students were enrolled. There were 69 students in the control group, and 76 students in the smart glasses group. There were 89 male students and 56 female students, as well as 25 5th-year medical students and 120 6th-year medical students. The distribution according to gender and grade was not significantly different between the control and smart glasses groups (Table 1).

### Numbers analyzed

All participants were included in each analysis. There was no loss between the pre- and post-intervention tests.

### Outcomes and estimation

*Medical students’ changes in scores for self-assessed efficacy in the smart glasses group were better than those in the control group.*

**Fig. 2.**
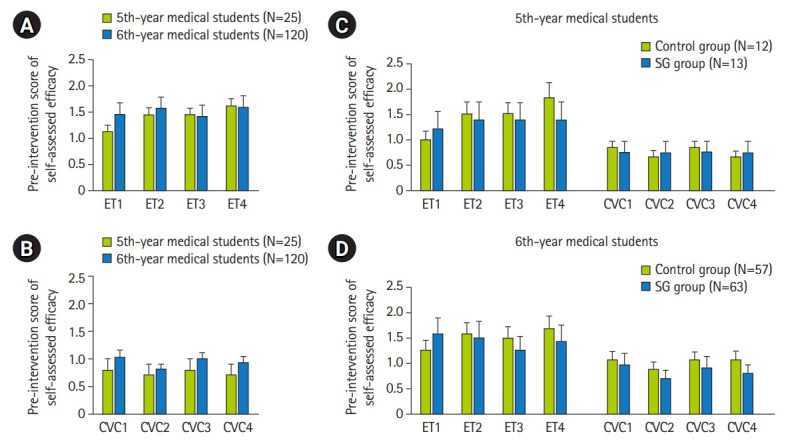
(A–D) The distribution of medical students’ pre-intervention scores of self-assessed efficacy in different groups. SG, smart glasses.

The pre-intervention self-assessed efficacy of 4 steps in the ETI and CVC procedures was not significantly different between 5th- and 6th-year medical students (Fig. 2A, B and Supplement 7). In both the 5th-year and 6th-year medical students, there was a trend for pre-intervention self-assessed efficacy in the control group to be higher than in the smart glasses group in most steps of the ETI and CVC procedures (Fig. 2C, D and Supplement 8).

**Fig. 3.**
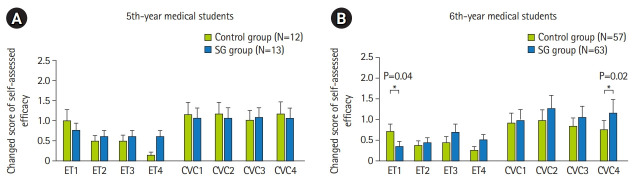
(A, B) The distribution of changes in medical students’ scores for self-assessed efficacy in different groups. SG, smart glasses. *P<0.05.

Most medical students reached a full score of self-assessed efficacy in each step of both the ETI and CVC procedures at the second assessment (Supplement 8, post-intervention scores). In both 5th-year and 6th-year medical students, there was a trend for the change in the self-assessed efficacy score to be higher in the smart glasses group than in the control group in most steps of the ETI and CVC procedures (Fig. 3A, B and Supplement 9), especially for 6th-year medical students in ET3, ET4, CVC2, CVC3, and CVC4 (ET3=“proper use of laryngoscope, without grinding the teeth,” ET4=“remove stylet and inflate the cuff,” CVC2=“insert the guide wire properly with sterile technique and appropriate depth,” CVC3=“place the skin dilator properly with appropriate depth,” and CVC4=“insert the catheter properly”) (Fig. 3B). Significance was noted for the statements of CVC4 and ET1, and an opposite trend was seen in the results for ET1(Fig. 3B). No significant difference in self-assessed learning efficacy according to gender was found in either the control or smart glasses group (Supplement 10).

*Sixth-year medical students in the smart glasses group showed higher satisfaction with the training tool as well as the instructor’s teaching and workshop than those in the control group.*

**Fig. 4.**
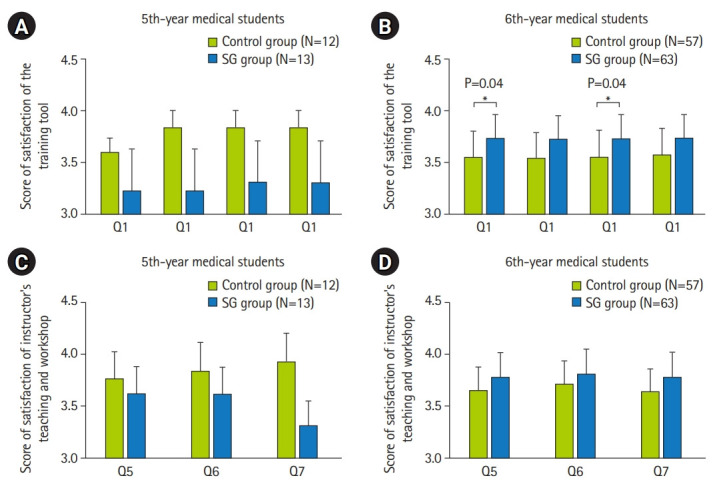
(A–D) The distribution of medical students’ satisfaction with the training-related statements in different groups. SG, smart glasses. *P<0.05.

In 6th-year medical students, the smart glasses group’s scores for satisfaction on training tool-related statements (Q1, Q2, Q3, and Q4 in Fig. 4B) and instructor’s teaching and workshop-related statements (Q5, Q6, and Q7 in Fig. 4D) were higher than those of participants in the control group (Supplement 11). Significance was noted for the statements of Q1 and Q3: Q1=“the training tool could provide accurate information for practice in a space-limited field,” Q3=“the training tool is interactive” (Fig. 4B) and Q5=“the instructor could teach students with the training tool properly,” Q6=“the instructor’s demonstration and practice are useful for clinical rotations,” Q7=“overall, I am satisfied with this workshop” (Fig. 4D). In the 5th-year medical students, there was a trend for the control group’s scores for satisfaction with training tool-related statements (Q1, Q2, Q3, and Q4 in Fig. 4A) and satisfaction with the instructor’s teaching and workshop-related statements (Q5, Q6, and Q7 in Fig. 4C) to be higher than those of participants in the smart glasses group. There was no significant difference in satisfaction scores in either the control or smart glasses group according to gender (Supplement 12).

### Adverse events

No adverse events were reportable.

## Discussion

### Key results

In this study, most students considered themselves competent to complete steps of these procedures after the second round of practice, and the improvement of self-assessed efficacy was higher in students with the usage of smart glasses. As for the satisfaction of students with smart glasses with the training, 6th-year medical students showed higher satisfaction, contrary to the responses from 5th-year medical students.

### Interpretation

Although medical students were more confident in performing ETI and CVC procedures after being familiar with smart glasses and receiving repetitive practice, there may be some inherent differences in learning ETI and CVC procedures via smart glasses between 5th- and 6th-year medical students.

For ETI and CVC procedures, we found that medical students often could not see the detailed practice of certain steps in these procedures, especially when the clinical environment was crowded. They could not see the operator’s point of view to explore the practice of certain steps related to anatomical positions. Even when the student was the operator, the instructors might not have been able to see the student’s point of view to determine problems in practice and provide real-time instructions.

As described by Chao et al. [8,9], the most common reasons for failure in ETI and CVC procedures of medical students were poor visualization of vocal cords, suboptimal placement of the laryngoscope in ETI, and the inability to find the vein and failure to pass the guidewire in CVC. These steps involve practices in space-limited fields, such as the opening of the mouth in ETI, and the sterile field of the puncture site in CVC. With smart glasses, students could practice on mannequins under the real-time supervision of instructors and see other operator’s practice in a first-person point of view through online video [10].

For 5th- and 6th-year medical students, the effectiveness of smart glasses in assisting their learning of ETI and CVC procedures was different. First, their clinical exposure differs. The 6th-year medical students had received 1 year of clinical rotations and had the experience of overnight shifts with the senior residents, which gave them more access to critical clinical conditions, in which ETI and CVC procedures were involved. Furthermore, their level of competency was different.

Sixth-year medical students may already have an awareness of the key difficult steps of these procedures after clinical exposure and having experienced failure. By contrast, 5th-year medical students are naïve to the clinical environment without real failure experience of ETT or CVC. Therefore, in our study, the 5th-year medical students with smart glasses showed lower satisfaction than the control group.

In this study, there was a trend for the pre-intervention self-assessed efficacy of students in the control group to be higher than that of the smart glasses group for most steps of ETI and CVC procedures (Fig. 2C, D and Supplement 8). However, in the second assessment (post-intervention score, Supplement 8), there was a trend for the changes in scores of self-assessed efficacy in the smart glasses group to be higher than in the control group in most steps of ETI and CVC procedures (Fig. 3A, B and Supplement 9). Instructors’ feedback mentioned the problem of adjusting the display by moving the head to ensure the recorded video was compatible with the student’s point of view. It is possible that in the students’ first practice with smart glasses, they were not familiar with smart glasses, which resulted in lower performance. However, by the second practice session, the benefits of smart glasses could be clearly observed. This problem could be resolved by providing instructions in advance on how to use the smart glasses.

A recent study revealed gender disparities in medical students’ procedural skills, such as ETI and CVC, during clerkship [11]. However, this effect was not seen in either the control or smart glasses group in our study. This may be explained by the fact that smart glasses were used in the simulation environment, rather than in actual clinical practice. Future research may explore the effects of medical students’ gender on the clinical practice of procedural skills using smart glasses.

### Limitations/generalizability

The assessment of student performance only included self-assessed efficacy, without objective evaluation by instructors. Previous experience with the procedure or usage of smart glasses should also be considered. In addition, this was a single-center pilot study, and many enrolled participants were 6th-year medical students. More 5th-year medical students should be enrolled to optimize the training workshop for 5th- and 6th-year medical students.

### Suggestions

We believe this study can facilitate future training in procedural skills using smart glasses in medical students.

### Conclusion

Smart glasses are a suitable simulation tool for training students to perform ETI and CVC procedures, improving self-assessed efficacy and increasing satisfaction with training, especially for procedural steps in a space-limited field. For junior students, training on procedures could be adjusted to reflect their limited clinical exposure. Before practicing skills with smart glasses, instructions on using new technology should precede to improve students’ satisfaction.

## Figures and Tables

**Table 1.** Baseline characteristics of medical students

Characteristic	No. of students (%)			P-value^a)^
	Control group (N=69)	Smart glasses group (N=76)	Total (N=145)	
Gender				0.57
Male	44 (64)	45 (59)	89	
Female	25 (36)	31 (41)	56	
Year				0.96
5th year	12 (17)	13 (17)	25	
6th year	57 (83)	63 (83)	120	

a) P-values were calculated using the Pearson chi-square test.

## Footnotes

**Data availability**
Data files are available from Harvard Dataverse: https://doi.org/10.7910/DVN/SEG7UY Dataset 1. Raw score data of self-assessment checklist and satisfaction questionnaire for each student.

## Supplementary materials

Supplementary files are available from Harvard Dataverse: https://doi.org/10.7910/DVN/SEG7UY

Supplement 1. Video of smart glasses practice.

Supplement 2. Self-assessment checklist for endotracheal intubation.

Supplement 3. Self-assessment checklist for central venous catheterization.

Supplement 4. Satisfaction questionnaire.

Supplement 5. Content validity of each statement in checklists.

Supplement 6. Content validity of each statement in satisfaction questionnaire.

Supplement 7. Basal characteristics of medical students.

Supplement 8. The distribution of 5th- and 6th-year medical students’ 1st score of self-assessed efficacy.

Supplement 9. The distribution of 5th- and 6th-year medical students’ 1st- and 2nd-score of self-assessed efficacy in control and SG groups.

Supplement 10. The distribution of 5th- and 6th-year medical students’ improving score of self-assessed efficacy in control and SG groups.

Supplement 11. The distribution of 5th- and 6th-year medical students’ satisfaction to the statements listed in the satisfaction questionnaire in control and SG groups.

Supplement 12. The distribution of medical students’ satisfaction to the statements listed in the satisfaction questionnaire in control and SG groups of different gender.

Supplement 13. Audio recording of the abstract.
