# Supplementary material for: Medical students’ self-assessed efficacy and satisfaction with training on endotracheal intubation and central venous catheterization with smart glasses in Taiwan: a non-equivalent control-group pre- and post-test study
Source: J Educ Eval Health Prof. 2022 Sep 2;19:25. doi: 10.3352/jeehp.2022.19.25 (PMC9681602; doi:10.3352/jeehp.2022.19.25)
Supplement: Supplementary file 3 — Supplement 2. Self-assessment checklist for endotracheal intubation. [file jeehp-19-25-suppl2.docx]

**Supplement 2.** Self-assessment checklist for endotracheal intubation

| Self-assessment checklist for endotracheal intubation |
| --- |
| **ET1.** Remove any denture, or foreign bodies in the oral cavity.  □ Could complete it □ Could not complete it |
| **ET2.** Maintain sniffing position, with preoxygenation by bag-valve mask.  □ Could complete it □ Could not complete it |
| **ET3.** Proper use of laryngoscope, without grinding the teeth.  □ Could complete it □ Could not complete it |
| **ET4.** Remove stylet and inflate the cuff.  □ Could complete it □ Could not complete it |
