# Supplementary material for: Medical students’ self-assessed efficacy and satisfaction with training on endotracheal intubation and central venous catheterization with smart glasses in Taiwan: a non-equivalent control-group pre- and post-test study
Source: J Educ Eval Health Prof. 2022 Sep 2;19:25. doi: 10.3352/jeehp.2022.19.25 (PMC9681602; doi:10.3352/jeehp.2022.19.25)
Supplement: Supplementary file 4 — Supplement 3. Self-assessment checklist for central venous catheterization. [file jeehp-19-25-suppl3.docx]

**Supplement 3.** Self-assessment checklist for central venous catheterization

| Self-assessment checklist for central venous catheterization |
| --- |
| **CVC1.** Choose puncture site, apply local anesthesia, and place the larger introducer needle.  □ Could complete it □ Could not complete it |
| **CVC2.** Insert the guide wire properly with sterile technique and appropriate depth.  □ Could complete it □ Could not complete it |
| **CVC3.** Place the skin dilator properly with appropriate depth.  □ Could complete it □ Could not complete it |
| **CVC4.** Insert the catheter properly.  □ Could complete it □ Could not complete it |
