# Supplementary material for: Medical students’ self-assessed efficacy and satisfaction with training on endotracheal intubation and central venous catheterization with smart glasses in Taiwan: a non-equivalent control-group pre- and post-test study
Source: J Educ Eval Health Prof. 2022 Sep 2;19:25. doi: 10.3352/jeehp.2022.19.25 (PMC9681602; doi:10.3352/jeehp.2022.19.25)
Supplement: Supplementary file 5 — Supplement 4. Satisfaction questionnaire. [file jeehp-19-25-suppl4.docx]

**Supplement 4.** Satisfaction questionnaire

| **Statements related to the satisfaction of the training tool** |
| --- |
| **Q1.** The training tool could provide accurate information of the practice in space-limited field.  □ very agree □ agree □ disagree □ very disagree |
| **Q2.** The training tool is convenient to use.  □ Very agree □ Agree □ Disagree □ Very disagree |
| **Q3.** The training tool is interactive.  □ Very agree □ Agree □ Disagree □ Very disagree |
| **Q4.** I am willing to use this training tool.  □ Very agree □ Agree □ Disagree □ Very disagree |
| **Statements related to the satisfaction of instructor’s teaching and workshop** |
| **Q5.** The instructor could teach students with the training tool properly.  □ Very agree □ Agree □ Disagree □ Very disagree |
| **Q6.** The instructor’s demonstration and practice are useful for clinical rotations.  □ Very agree □ Agree □ Disagree □ Very disagree |
| **Q7.** Overall, I am satisfactory to this workshop.  □ Very agree □ Agree □ Disagree □ Very disagree |
