# Supplementary material for: Medical students’ self-assessed efficacy and satisfaction with training on endotracheal intubation and central venous catheterization with smart glasses in Taiwan: a non-equivalent control-group pre- and post-test study
Source: J Educ Eval Health Prof. 2022 Sep 2;19:25. doi: 10.3352/jeehp.2022.19.25 (PMC9681602; doi:10.3352/jeehp.2022.19.25)
Supplement: Supplementary file 6 — Supplement 5. Content validity of each statement in checklists. [file jeehp-19-25-suppl5.docx]

**Supplement 5.** Content validity of each statement in checklists

| Statements of checklists of ETI and CVC | Content validity index viewed by each expert | | | | Average |
| --- | --- | --- | --- | --- | --- |
|  | Expert 1 | Expert 2 | Expert 3 | Expert 4 |  |
| ETI1: remove any denture, or foreign bodies in the oral cavity. | 0.8 | 0.9 | 0.7 | 0.8 | 0.8 |
| ETI2: maintain sniffing position, with preoxygenation by bag-valve mask. | 0.7 | 0.9 | 0.8 | 0.9 | 0.83 |
| ETI3: proper use of laryngoscope, without grinding the teeth. | 0.7 | 0.8 | 0.7 | 0.9 | 0.78 |
| ETI4: remove stylet and inflate the cuff. | 0.8 | 0.9 | 0.8 | 0.7 | 0.8 |
| CVC1: choose puncture site, apply local anesthesia, and place the larger introducer needle. | 0.9 | 0.8 | 0.7 | 0.8 | 0.8 |
| CVC2: insert the guide wire properly with sterile technique and appropriate depth. | 0.8 | 0.8 | 0.9 | 0.7 | 0.8 |
| CVC3: place the skin dilator properly with appropriate depth. | 0.8 | 0.9 | 0.9 | 0.7 | 0.83 |
| CVC4: insert the catheter properly. | 0.9 | 0.8 | 0.7 | 0.9 | 0.83 |
| Intraclass correlation coefficients | 0.78 | | | | |

ETI, endotracheal intubation; CVC, central venous catheterization.
