# Supplementary material for: Medical students’ self-assessed efficacy and satisfaction with training on endotracheal intubation and central venous catheterization with smart glasses in Taiwan: a non-equivalent control-group pre- and post-test study
Source: J Educ Eval Health Prof. 2022 Sep 2;19:25. doi: 10.3352/jeehp.2022.19.25 (PMC9681602; doi:10.3352/jeehp.2022.19.25)
Supplement: Supplementary file 7 — Supplement 6. Content validity of each statement in satisfaction questionnaire. [file jeehp-19-25-suppl6.docx]

**Supplement 6.** Content validity of each statement in satisfaction questionnaire

| Statements of satisfaction questionnaire | Content validity index viewed by each expert | | | | Average |
| --- | --- | --- | --- | --- | --- |
|  | Expert 1 | Expert 2 | Expert 3 | Expert 4 |  |
| **Q1.** The training tool could provide accurate information of the practice in space-limited field | 0.9 | 0.7 | 0.8 | 0.9 | 0.83 |
| **Q2.** The training tool is convenient to use. | 0.7 | 0.8 | 0.7 | 0.9 | 0.78 |
| **Q3.** The training tool is interactive | 0.8 | 0.9 | 0.9 | 0.8 | 0.85 |
| **Q4.** I am willing to use this training tool. | 0.8 | 0.9 | 0.7 | 0.7 | 0.9 |
| **Q5.** The instructor could teach students with the training tool properly. | 0.7 | 0.7 | 0.9 | 0.9 | 0.78 |
| **Q6.** The instructor’s demonstration and practice are useful for clinical rotations | 0.8 | 0.9 | 0.9 | 0.8 | 0.9 |
| **Q7.** Overall, I am satisfactory to this workshop. | 0.9 | 0.7 | 0.9 | 0.7 | 0.8 |
| Intraclass correlation coefficients | 0.75 | | | | |
