# Supplementary material for: Medical students’ self-assessed efficacy and satisfaction with training on endotracheal intubation and central venous catheterization with smart glasses in Taiwan: a non-equivalent control-group pre- and post-test study
Source: J Educ Eval Health Prof. 2022 Sep 2;19:25. doi: 10.3352/jeehp.2022.19.25 (PMC9681602; doi:10.3352/jeehp.2022.19.25)
Supplement: Supplementary file 8 — Supplement 7. Basal characteristics of medical students. [file jeehp-19-25-suppl7.docx]

**Supplement 7.** Basal characteristics of medical students

| Group | Control group (N=69) | SG group (N=76) | Total (N=145) | P-value^a)^ |
| --- | --- | --- | --- | --- |
| Gender |  |  |  | 0.57 |
| Male | 44 (64) | 45 (59) | 89 |  |
| Female | 25 (36) | 31 (41) | 56 |  |
| Grade |  |  |  | 0.96 |
| 5th year | 12 (17) | 13 (17) | 25 |  |
| 6th year | 57 (83) | 63 (83) | 120 |  |

Values are presented as number (%).

SG, smart glasses.

^a)^P-values were calculated with use of Pearson’s chi-square test.
