# Supplementary material for: Medical students’ self-assessed efficacy and satisfaction with training on endotracheal intubation and central venous catheterization with smart glasses in Taiwan: a non-equivalent control-group pre- and post-test study
Source: J Educ Eval Health Prof. 2022 Sep 2;19:25. doi: 10.3352/jeehp.2022.19.25 (PMC9681602; doi:10.3352/jeehp.2022.19.25)
Supplement: Supplementary file 9 — Supplement 8. The distribution of 5th- and 6th-year medical students’ 1st score of self-assessed efficacy. [file jeehp-19-25-suppl8.docx]

**Supplement 8.** The distribution of 5th- and 6th-year medical students’ 1st score of self-assessed efficacy

| Statements of checklists | 1st score of self-assessed efficacy | | |
| --- | --- | --- | --- |
|  | 5th-year medical students (N=25) | 6th-year medical students (N=120) | P-value |
| No. in ETI |  |  |  |
| ET1 | 1.12±1.01 (0.70–1.54) | 1.47±1.31 (1.31–1.63) | 0.08 |
| ET2 | 1.44±0.92 (1.06–1.82) | 1.58±1.44 (1.44–1.73) | 0.43 |
| ET3 | 1.44±0.92 (1.06–1.82) | 1.42±1.25 (1.25–1.58) | 0.90 |
| ET4 | 1.60±0.82 (1.26–1.94) | 1.60±1.45 (1.45–1.75) | 1.00 |
| No. in CVC |  |  |  |
| CVC1 | 0.80±1.00 (0.39–1.21) | 1.05±0.87 (0.87–1.23) | 0.25 |
| CVC2 | 0.72±0.98 (0.32–1.12) | 0.82±0.64 (0.64–1.00) | 0.65 |
| CVC3 | 0.80±1.00 (0.39–1.21) | 1.02±0.84 (0.84–1.20) | 0.32 |
| CVC4 | 0.72±0.98 (0.32–1.12) | 0.95±0.77 (0.77–1.13) | 0.29 |

Values are presented as mean score±standard deviation (95% confidence interval).

ETI, endotracheal intubation; CVC, central venous catheterization.
