# Supplementary material for: Medical students’ self-assessed efficacy and satisfaction with training on endotracheal intubation and central venous catheterization with smart glasses in Taiwan: a non-equivalent control-group pre- and post-test study
Source: J Educ Eval Health Prof. 2022 Sep 2;19:25. doi: 10.3352/jeehp.2022.19.25 (PMC9681602; doi:10.3352/jeehp.2022.19.25)
Supplement: Supplementary file 10 — Supplement 9. The distribution of 5th- and 6th-year medical students’ 1st- and 2nd-score of self-assessed efficacy in control and SG groups. [file jeehp-19-25-suppl9.docx]

**Supplement 9.** The distribution of 5th- and 6th-year medical students’ 1st- and 2nd-score of self-assessed efficacy in control and SG groups

| Statements of checklists | 1st score of self-assessed efficacy | | | | | | 2nd score^a)^ |
| --- | --- | --- | --- | --- | --- | --- | --- |
|  | 5th-year medical students | | P-value | 6th-year medical students | | P-value | Overall (N=145) |
|  | Control group (N=12) | SG group (N=13) |  | Control group (N=57) | SG group (N=63) |  |  |
| No. in ETI |  |  |  |  |  |  |  |
| ET1 | 1.00±1.04 (0.34–1.66) | 1.23±1.01 (0.62–1.84) | 0.58 | 1.3±0.96 (1.04–1.55) | 1.62±0.79 (1.42–1.82) | 0.04 | 2.00^b)^ |
| ET2 | 1.50±0.90 (0.93–2.07) | 1.38±0.96 (0.80–1.97) | 0.76 | 1.61±0.80 (1.40–1.83) | 1.56±0.84 (1.34–1.77) | 0.69 | 2.00^b)^ |
| ET3 | 1.50±0.90 (0.93–2.07) | 1.38±0.96 (0.80–1.97) | 0.76 | 1.54±0.85 (1.32–1.77) | 1.30±0.96 (1.06–1.54) | 0.14 | 2.00^b)^ |
| ET4 | 1.83±0.58 (1.47–2.20) | 1.38±0.96 (0.80–1.97) | 0.17 | 1.72±0.70 (1.53–1.91) | 1.49±0.88 (1.27–1.71) | 0.12 | 2.00^b)^ |
| No. in CVC |  |  |  |  |  |  |  |
| CVC1 | 0.83±1.03 (0.18–1.49) | 0.77±1.01 (0.16–1.38) | 0.87 | 1.09±1.01 (0.82–1.35) | 1.02±1.01 (0.76–1.27) | 0.69 | 1.99±0.17 (1.96–2.01) |
| CVC2 | 0.67±0.98 (0.04–1.29) | 0.77±1.01 (0.16–1.38) | 0.80 | 0.91±1.01 (0.65–1.18) | 0.73±0.97 (0.49–0.97) | 0.31 | 1.93±0.37 (1.87–1.99) |
| CVC3 | 0.83±1.03 (0.18–1.49) | 0.77±1.01 (0.16–1.38) | 0.87 | 1.09±1.01 (0.82–1.35) | 0.95±1.01 (0.70–1.21) | 0.46 | 1.94±0.33 (1.89–2.00) |
| CVC4 | 0.67±0.98 (0.04–1.29) | 0.77±1.01 (0.16–1.38) | 0.80 | 1.09±1.01 (0.82–1.35) | 0.83±0.99 (0.58–1.08) | 0.15 | 1.92±0.40 (1.85–1.98) |

Values are presented as mean score±standard deviation (95% confidence interval).

SG, smart glasses; ETI, endotracheal intubation; CVC, central venous catheterization.

^a)^Score of self-assessed efficacy. ^b)^All data were 2.00.
