# Supplementary material for: Medical students’ self-assessed efficacy and satisfaction with training on endotracheal intubation and central venous catheterization with smart glasses in Taiwan: a non-equivalent control-group pre- and post-test study
Source: J Educ Eval Health Prof. 2022 Sep 2;19:25. doi: 10.3352/jeehp.2022.19.25 (PMC9681602; doi:10.3352/jeehp.2022.19.25)
Supplement: Supplementary file 11 — Supplement 10. The distribution of 5th- and 6th-year medical students’ improving score of self-assessed efficacy in control and SG groups. [file jeehp-19-25-suppl10.docx]

**Supplement 10.** The distribution of 5th- and 6th-year medical students’ improving score of self-assessed efficacy in control and SG groups

| Statements of checklists | Improving score of self-assessed efficacy | | | | | |
| --- | --- | --- | --- | --- | --- | --- |
|  | 5th-year medical students | | P-value | 6th-year medical students | | P-value |
|  | Control group (N=12) | SG group (N=13) |  | Control group (N=57) | SG group (N=63) |  |
| No. in ETI |  |  |  |  |  |  |
| ET1 | 1.00±1.04 (0.34 to 1.66) | 0.77±1.01 (0.16 to1.38) | 0.58 | 0.70±0.96 (0.45 to 0.96) | 0.38±0.79 (0.18 to 0.58) | 0.0477 |
| ET2 | 0.50±0.90 (-0.07 to 1.07) | 0.62±0.96 (0.03 to 1.20) | 0.76 | 0.39±0.80 (0.17 to0.60) | 0.44±0.84 (0.23 to 0.66) | 0.6966 |
| ET3 | 0.50±0.90 (-0.07 to 1.07) | 0.62±0.96 (0.03 to 1.20) | 0.76 | 0.46±0.85 (0.23 to 0.68) | 0.70±0.96 (0.46 to 0.94) | 0.1473 |
| ET4 | 0.17±0.58 (-0.20 to 0.53) | 0.62±0.96 (0.03 to 1.20) | 0.17 | 0.28±0.70 (0.09 to 0.47) | 0.51±0.88 (0.29 to 0.73) | 0.1223 |
| No. in CVC |  |  |  |  |  |  |
| CVC1 | 1.17±1.03 (0.51 to 1.82) | 1.08±1.04 (0.45 to 1.70) | 0.83 | 0.91±1.01 (0.65 to 1.18) | 0.98±1.01 (0.73 to 1.24) | 0.69 |
| CVC2 | 1.17±1.03 (0.51 to 1.82) | 1.08±1.04 (0.45 to 1.70) | 0.83 | 0.98±1.01 (0.71 to 1.25) | 1.27±0.97 (1.03 to 1.51) | 0.11 |
| CVC3 | 1.00±1.04 (0.34 to 1.66) | 1.08±1.04 (0.45 to 1.70) | 0.85 | 0.84±1.00 (0.58 to 1.11) | 1.05±1.01 (0.79 to 1.30) | 0.26 |
| CVC4 | 1.17±1.03 (0.51 to 1.82) | 1.08±1.04 (0.45 to 1.70) | 0.83 | 0.77±0.98 (0.51 to 1.03) | 1.17±0.99 (0.92 to 1.42) | 0.02 |

Values are presented as mean score±standard deviation (95% confidence interval).

SG, smart glasses; ETI, endotracheal intubation; CVC, central venous catheterization.
