# Supplementary material for: Medical students’ self-assessed efficacy and satisfaction with training on endotracheal intubation and central venous catheterization with smart glasses in Taiwan: a non-equivalent control-group pre- and post-test study
Source: J Educ Eval Health Prof. 2022 Sep 2;19:25. doi: 10.3352/jeehp.2022.19.25 (PMC9681602; doi:10.3352/jeehp.2022.19.25)
Supplement: Supplementary file 12 — Supplement 11. The distribution of 5th- and 6th-year medical students’ satisfaction to the statements listed in the satisfaction questionnaire in control and SG groups. [file jeehp-19-25-suppl11.docx]

**Supplement 11.** The distribution of 5th- and 6th-year medical students’ satisfaction to the statements listed in the satisfaction questionnaire in control and SG groups

| Statements of questionnaire | Satisfaction regarding learning experience | | | | | |
| --- | --- | --- | --- | --- | --- | --- |
|  | 5th-year medical students | | P-value | 6th-year medical students | | P-value |
|  | Control group (N=12) | SG group (N=13) |  | Control group (N=57) | SG group (N=63) |  |
| Statements related to the satisfaction of training tool |  |  |  |  |  |  |
| Q1 | 3.58±0.90 (3.01–4.16) | 3.23±1.09 (2.57–3.89) | 0.38 | 3.54±0.54 (3.40–3.69) | 3.73±0.45 (3.62–3.84) | 0.04 |
| Q2 | 3.83±0.39 (3.59–4.08) | 3.23±1.17 (2.53–3.94) | 0.09 | 3.53±0.63 (3.36–3.69) | 3.71±0.49(3.59–3.84) | 0.06 |
| Q3 | 3.83±0.39 (3.59–4.08) | 3.31±1.11 (2.64–3.98) | 0.12 | 3.54±0.57 (3.39–3.69) | 3.73±0.45 (3.62–3.84) | 0.04 |
| Q4 | 3.83±0.39 (3.59–4.08) | 3.31±1.11 (2.64–3.98) | 0.12 | 3.56±0.54 (3.42–3.70) | 3.73±0.45 (3.62–3.84) | 0.06 |
| Statements related to the satisfaction of instructor’s teaching and workshop |  |  |  |  |  |  |
| Q5 | 3.75±0.45 (3.46–4.04) | 3.62±0.51 (3.31–3.92) | 0.49 | 3.65±0.48 (3.52–3.78) | 3.78±0.42 (3.67–3.88) | 0.12 |
| Q6 | 3.83±0.39 (3.59–4.08) | 3.62±0.51 (3.31–3.92) | 0.24 | 3.70±0.46 (3.58–3.82) | 3.81±0.40 (3.71–3.91) | 0.17 |
| Q7 | 3.92±0.29 (3.73–4.10) | 3.31±1.11 (2.64–3.98) | 0.07 | 3.63±0.49 (3.50–3.76) | 3.78±0.42 (3.67–3.88) | 0.07 |

Values are presented as mean score±standard deviation (95% confidence interval).

SG, smart glasses; ETI, endotracheal intubation; CVC, central venous catheterization.
