# Supplementary material for: Medical students’ self-assessed efficacy and satisfaction with training on endotracheal intubation and central venous catheterization with smart glasses in Taiwan: a non-equivalent control-group pre- and post-test study
Source: J Educ Eval Health Prof. 2022 Sep 2;19:25. doi: 10.3352/jeehp.2022.19.25 (PMC9681602; doi:10.3352/jeehp.2022.19.25)
Supplement: Supplementary file 13 — Supplement 12. The distribution of medical students’ satisfaction to the statements listed in the satisfaction questionnaire in control and SG groups of different gender. [file jeehp-19-25-suppl12.docx]

**Supplement 12.** The distribution of medical students’ satisfaction to the statements listed in the satisfaction questionnaire in control and SG groups of different gender

| Statements of questionnaire | Satisfaction regarding learning experience | | | | | |
| --- | --- | --- | --- | --- | --- | --- |
|  | Control group (N=69) | | P-value | SG group (N=76) | | P-value |
|  | Female (N=25) | Male (N=44) |  | Female (N=31) | Male (N=45) |  |
| Statements related to the satisfaction of training tool |  |  |  |  |  |  |
| Q1 | 3.48±0.51 (3.27–3.69) | 3.59±0.66 (3.39–3.79) | 0.46 | 3.61±0.50 (3.43–3.79) | 3.67±0.71 (3.45–3.88) | 0.69 |
| Q2 | 3.60±0.50 (3.39–3.81) | 3.57±0.66 (3.37–3.77) | 0.83 | 3.61±0.56 (3.41–3.82) | 3.64±0.74 (3.42–3.87) | 0.84 |
| Q3 | 3.56±0.51 (3.35–3.77) | 3.61±0.58 (3.44–3.79) | 0.70 | 3.65±0.49 (3.47–3.82) | 3.67±0.71 (3.45–3.88) | 0.87 |
| Q4 | 3.64±0.49 (3.44–3.84) | 3.59±0.54 (3.43–3.76) | 0.70 | 3.65±0.49 (3.47–3.82) | 3.67±0.71 (3.45–3.88) | 0.87 |
| Statements related to the satisfaction of instructor’s teaching and workshop |  |  |  |  |  |  |
| Q5 | 3.64±0.49 (3.44–3.84) | 3.68±0.47 (3.54–3.83) | 0.72 | 3.71±0.46 (3.54–3.88) | 3.78±0.42 (3.65–3.90) | 0.50 |
| Q6 | 3.68±0.48 (3.48–3.88) | 3.75±0.44 (3.62–3.88) | 0.53 | 3.74±0.44 (3.58–3.91) | 3.80±0.40 (3.68–3.92) | 0.55 |
| Q7 | 3.64±0.49 (3.44–3.84) | 3.70±0.46 (3.56–3.84) | 0.58 | 3.68±0.48 (3.50–3.85) | 3.71±0.69 (3.50–3.92) | 0.80 |

Values are presented as mean score±standard deviation (95% confidence interval).

SG, smart glasses; ETI, endotracheal intubation; CVC, central venous catheterization.
